# The Effectiveness of Different Diet Strategies to Reduce Type 2 Diabetes Risk in Youth

**DOI:** 10.3390/nu8080486

**Published:** 2016-08-09

**Authors:** Megan L. Gow, Sarah P. Garnett, Louise A. Baur, Natalie B. Lister

**Affiliations:** 1 The Children’s Hospital at Westmead, Westmead 2145, Australia; sarah.garnett@health.nsw.gov.au (S.P.G.); Louise.Baur@health.nsw.gov.au (L.A.B.); natalie.lister@health.nsw.gov.au (N.B.L.); 2 The Discipline of Child and Adolescent Health, The University of Sydney, Westmead 2145, Australia

**Keywords:** type 2 diabetes, obesity, prevention, child, adolescent, youth, carbohydrate, diet intervention

## Abstract

Type 2 diabetes in children and adolescents has become a prominent clinical issue in recent decades. Increasing numbers of young people have risk factors for type 2 diabetes, particularly obesity, indicating the need for effective type 2 diabetes prevention strategies. The aim of this review was to identify specific dietary strategies that optimize improvements in risk factors for type 2 diabetes in youth and hence reduce the risk of type 2 diabetes development. Our review of the current literature indicates that dietary interventions lead to weight loss when intervention adherence is high. However, in addition to weight loss, a diet that is reduced in carbohydrates may optimize improvements in other type 2 diabetes risk factors, including insulin resistance and hyperglycemia. While further research is needed to confirm this finding, reduced carbohydrate diets may include a very low-carbohydrate diet, a very low-energy diet, a lower-glycemic-index diet, and/or an intermittent fasting diet. This array of dietary strategies provides a suite of intervention options for clinicians to recommend to young people at risk of type 2 diabetes. However, these findings are in contrast to current guidelines for the prevention of type 2 diabetes in adults which recommends a low-fat, high-carbohydrate diet.

## 1. Introduction

Increases in obesity rates around the world over the last several decades have preceded the emergence of type 2 diabetes (T2DM) in children, adolescents, and young adults as an important clinical issue; now affecting up to 5.3% of 0–19 years old in certain populations [1,2]. In the past decade, research has demonstrated the aggressive nature of T2DM in young people, including the early development of diabetes-related complications which ultimately lead to premature mortality [3,4,5,6,7]. These factors highlight the importance of developing effective prevention strategies.

To the best of our knowledge, there have been no interventions conducted in young people with the primary end point being a reduction in T2DM incidence. This type of study would be difficult to undertake due to the number of children and adolescents who develop T2DM and the potentially long follow-up that would be required to detect an incidence reduction.

However, several risk factors for T2DM are modifiable, including obesity, features of the metabolic syndrome (including abdominal obesity, elevated triglycerides, low high density lipoprotein cholesterol (HDL-C), high blood pressure, and elevated plasma glucose [8]), insulin resistance, sedentary behaviour, and the intrauterine environment. Targeting these factors should facilitate a reduction in the risk of T2DM. A number of intervention studies conducted in children and adolescents aim to reduce obesity and concomitantly measure changes in other important modifiable risk factors, including insulin resistance and features of the metabolic syndrome. Such trials can indicate important strategies for reducing the risk of T2DM in young people.

In this review we aim to identify specific dietary patterns and/or macronutrient distributions that optimise improvements in prominent modifiable risk factors for T2DM, i.e., obesity, insulin resistance, and features of the metabolic syndrome in youth at increased T2DM risk, i.e., youth with at least one modifiable T2DM risk factor. Identification of optimal strategies may guide clinicians in their dietary prescription for youth at increased risk of T2DM.

## 2. Systematic Reviews of Dietary Interventions for Obesity Treatment

When aiming to reduce the risk of a young person developing T2DM, a dietary intervention will often aim to contribute to an energy deficit to improve weight status, which is the leading contributor to T2DM. Systematic reviews of randomised controlled trials (RCTs) of obesity treatment in children and adolescents acknowledge that lifestyle interventions incorporating a dietary component are effective (i.e., result in weight loss) up to 12 months from baseline [9,10,11]. The 2013 systematic review by Ho and colleagues demonstrated a reduction in body mass index (BMI) of −1.25 kg/m^2^, with 95% confidence interval (CI) −2.18 to −0.32 from lifestyle interventions compared with no treatment controls and also reported that, compared with usual care, lifestyle interventions led to significant improvements in fasting insulin, insulin resistance, blood lipids, and blood pressure [9]. The Stop/Traffic Light and caloric restriction approaches were the most commonly reported dietary interventions in that systematic review.

While systematic reviews highlighted the importance of dietary intervention for the treatment of obesity, they did not investigate the potential advantage of any particular dietary pattern and/or macronutrient distribution for weight loss and improving other T2DM risk factors.

## 3. Macronutrient Distribution of the Diet and Weight Loss

There has been much interest in recent years on whether there is an optimal dietary macronutrient distribution for weight loss, particularly in adults. Our 2014 systematic review with meta-analysis examined, for the first time, whether variations in dietary macronutrient content differentially affect changes in weight and cardio-metabolic risk factors in children and adolescents who are overweight or obese [12]. Overall, the review indicated improved weight status in children and adolescents undergoing obesity treatment, irrespective of the macronutrient distribution of a reduced-energy diet.

## 4. Macronutrient Distribution of the Diet and Improvements in T2DM Risk Factors

While there may not be an impact on weight loss, findings from our 2014 systematic review did suggest the potential for the specific application of certain macronutrient distributions to target certain T2DM risk factors [12].

### 4.1. Very Low-Carbohydrate vs. “Traditional” Low-Fat, High-Carbohydrate Diet

Conventionally, a “low-fat” diet approach (typically aiming for approximately 45%–65% of daily energy as carbohydrate, <35% as fat and ~15% as protein, Table 1) has been utilised in child and adolescent obesity treatment programs, including the Stop/Traffic Light diet [13,14,15] and a standard caloric restriction approach [16,17,18]. Large T2DM prevention studies in adults demonstrate that a low-fat, high-carbohydrate diet is effective in the prevention of T2DM, reporting reductions in T2DM incidence of 58% after approximately 3 years of intervention and follow-up [19,20,21,22,23]. Conversely, this low-fat, high-carbohydrate diet approach has been postulated to promote hyperglycaemia and compensatory hyperinsulinemia which may lead to more rapid progression of T2DM in susceptible individuals [24]. It has been suggested that alternative dietary patterns and macronutrient distributions may facilitate prevention of T2DM by reducing postprandial glucose and insulin levels, improving glucose and lipid metabolism, and preserving β cell function [24,25].

A popular alternative to the low-fat diet is a very low-carbohydrate diet (typically aiming for <50 g carbohydrate per day, Table 1) with high or ad libitum fat and/or protein intakes (e.g., the Atkins diet). In the 2014 systematic review examining the effect of varying the macronutrient distribution of a reduced energy diet in the treatment of child and adolescent obesity [12], three of the included studies reported improved insulin levels and/or insulin resistance in the very low-carbohydrate group compared with the low-fat group immediately following active treatment [27,28] or at follow-up [29], described in Table 2. Studies demonstrating an advantage of the very low-carbohydrate diet generally had a better methodological quality score and larger sample size and were more recently conducted studies compared with studies which found no differences between diet groups. Although results were not consistent, findings suggest that there may be a particular benefit of a very low-carbohydrate diet in facilitating improvements in hyperinsulinemia as part of obesity treatment compared with a traditional low-fat approach in children and adolescents with obesity, at least in the short-term.

### 4.2. Increased-Protein vs. “Traditional” Standard-Protein, (Low-Fat) Diet

Another popular alternative to a conventional low-fat approach is a moderate-carbohydrate, increased-protein diet (typically aiming for approximately 26%–44% of daily energy as carbohydrates, <35% as fat and 20%–40% as protein). Increased-protein diets are posited to lead to greater weight loss by evoking sustained satiety despite negative energy balance, and sustaining energy expenditure despite loss in body mass by sparing loss of fat free mass [30].

In our 2014 systematic review examining the effect of varying the macronutrient distribution of a reduced-energy diet for the treatment of child and adolescent obesity, six studies compared an increased-protein and a “traditional” standard-protein diet. Most studies found improvements following the intervention for weight, fasting glucose, fasting insulin, insulin sensitivity, blood lipids, and blood pressure, but no study found any advantage of either diet [12]. Since this review, two further studies have been published reporting similar findings [31,32].

In adults, a 2012 systematic review of 24 trials demonstrated that an increased-protein diet compared to an isocaloric standard-protein diet can produce greater reduction in weight (−0.79 kg; 95% CI: −1.50, −0.08 kg), fat mass (−0.87 kg; 95% CI: −1.26, −0.48 kg), and triglycerides (−0.23 mmol/L; 95% CI: −0.33, −0.12 mmol/L), with better preservation of fat free mass and resting energy expenditure [33]. However, changes in fasting plasma glucose, fasting insulin, blood pressure, total cholesterol, low density lipoprotein cholesterol (LDL-C), and HDL-C were similar across dietary treatments [33].Together, these findings suggest that increasing the protein content of a reduced-energy diet, without a concurrent severe restriction on carbohydrate intake, does not affect the ability of a reduced energy diet to prevent the development of T2DM, compared with a standard low-fat diet approach.

## 5. Glycemic Index of the Diet

The glycemic index (GI) of carbohydrates is also an important dietary factor to consider. A diet that is lower in GI generally refers to a balanced diet that incorporates carbohydrate foods which are of a reduced glycemic load, i.e., foods/meals that produce a slower rise in blood glucose levels and have lower overall carbohydrate content [40]. Intervention studies in adults and children demonstrate that diets aiming for a lower-GI are safe and effective for improving insulin secretion, insulin resistance, body weight, and body composition [41,42,43,44,45]. A six month RCT in children with obesity comparing a lower-GI (GI: 60) versus higher-GI (GI: 90) hypocaloric diet found that waist circumference, BMI *z* score, and insulin resistance were significantly reduced in the lower-GI compared with the higher-GI diet group [34]. In another RCT, 26 children were randomly assigned to either a hypocaloric lower-GI (GI: 60) or a hypocaloric higher-GI (GI: 90) diet. After six months, insulin resistance was significantly reduced only in the lower-GI diet group (homeostatic model assessment of insulin resistance (HOMA-IR); higher-GI, baseline: 3.2 ± 1.6, 6 months: 3.2 ± 1.8, *p* = 0.98; lower-GI, baseline: 3.1 ± 1.5, 6 months: 2.4 ± 1.1, *p* = 0.04) [35]. In another RCT, consumption of a higher glycaemic load diet was associated with less weight loss even when adjusted for sex and pubertal status (*R*^2^ = 0.11, *p* = 0.007) [36]. However, the association was no longer significant when adjusted for total energy intake of the diet [36].

The Diet, Obesity and Genes (DiOGenes) study is the largest study conducted to date to examine the effect of varying the GI *and* protein content of a diet on weight and cardio-metabolic outcomes in children, recruiting families from eight European countries. Eligible parents were randomised as a family unit to one of five ad libitum diets: low-protein and low-GI; low-protein and high-GI; high-protein and low-GI; high-protein and high-GI; and control diet (national dietary guidelines; medium protein content and no instructions on GI) [46]. A difference of 15 GI units between the high-GI and low-GI diets was targeted [46]. The results of this study showed that neither GI nor protein had an isolated effect on body composition among children following an ad libitum diet. However, the low-protein, high-GI combination increased body fat, whereas the high-protein, low-GI combination was protective against obesity [47].

Cardio-metabolic risk factors, including fasting glucose, fasting insulin, and HOMA-IR, were assessed in 253 of the 817 children in the DiOGenes study. In this sub-sample, the children following a high-protein diet had significant reductions in waist circumference and serum LDL cholesterol compared with the low-protein diets [37]. No effect of GI was observed in this sub-sample. However, in the children who were deemed to have had high adherence to their intervention, waist circumference (*p* = 0.004), diastolic blood pressure (*p* = 0.007), mean arterial pressure (*p* = 0.005), fasting insulin (*p* = 0.013), and HOMA-IR (*p* = 0.016) were reduced in the high-protein compared with the low-protein diets, and serum insulin and insulin resistance were reduced in the lower-GI compared with the higher-GI diets (*p* = 0.04) [37]. Of note, the children in the DiOGenes study were children of parents who were overweight or obese and also included healthy weight children who were not necessarily at increased risk of developing T2DM. The children were not given advice on weight loss but were educated on the diet’s ability to regulate appetite [47].

## 6. Very Low-Energy Diet

A very low-energy diet (VLED) is a non-conventional dietary approach that has gained popularity due to its association with rapid weight loss. It is a very strict diet aiming for <800 kcal/day. VLEDs are largely protein based, and contain essential fatty acids, vitamins, and minerals, but very little carbohydrates (typically < 50 g), and are aimed at inducing ketosis [48]. They reduce portion size and, consequently, energy intake. Because it is considered to be so difficult to follow, a VLED is usually implemented short-term, aiming for rapid weight loss, and is comprised of meal replacement products (e.g., shakes, bars, soups, or desserts) to achieve a nutritionally adequate diet.

Studies in adolescents with obesity have demonstrated that a VLED can safely induce rapid weight loss in the short-term (6 to 15 kg over 3 to 12 weeks), while preserving lean body mass [38,39,49]. Studies have also demonstrated improvements in blood pressure, total cholesterol, HDL-C, LDL-C, triglycerides, fasting insulin, fasting glucose, glycosylated haemoglobin (HbA1c), and insulin sensitivity [38,39,49]. One of these studies found that a short-term (10 weeks) daily VLED (600–800 kcal/day) compared with a hypocaloric low-fat diet produced significantly greater reductions in percentage overweight and body fat (%) while maintaining fat free mass [38]. In another study comparing a VLED with a hypocaloric low-fat diet, weight loss was greater in the VLED group at 4 months but this was not sustained at 12 months [39]. That study did not demonstrate any differences in cardio-metabolic outcomes, including insulin, insulin resistance, and glucose levels at any time point [39]. In one other study, a daily VLED for a mean of 60 days was effective in decreasing the BMI and improving HbA1c in adolescents with obesity and T2DM [49].

Findings indicate that VLEDs are tolerated by adolescents and result in rapid weight loss, improvements in body composition, and improved metabolic risk profile, but sustainability of long-term results is not clear. The diet, although strict, may be an alternative to pharmacological therapies or surgical interventions to treat adolescents with severe obesity. However, VLEDs require intensive monitoring by a team of health professionals and it is unclear whether they convey a particular benefit on cardio-metabolic outcomes beyond advantages from weight loss.

## 7. Intermittent Modified Fasting

Daily modest caloric restriction can be difficult to sustain and it may be very difficult to adhere to a VLED. Therefore, a viable alternative may be intermittent modified fasting, popularised as the 5:2 diet, which has gained recent media interest and celebrity endorsement. This diet regimen typically includes one to four “fasting” (or VLED) days per week, where energy intake is drastically limited (typically less than 600 kcal), and three to six “feeding” days per week, where food is either consumed ad libitum or a diet based on healthy eating guidelines is prescribed. It is possible that intermittent modified fasting comprised of shorter periods of energy restriction coupled with longer periods of habitual energy intake may be more sustainable and promote better adherence than continuous daily energy restriction [50].

In adults, there is evidence that intermittent fasting is effective in the short-term (eight weeks to six months) to help individuals with obesity lose body weight (4%–8%) and body fat, and improve insulin sensitivity and other risk factors for T2DM [50,51,52,53,54,55,56,57,58,59,60]. Certain studies demonstrate that intermittent fasting may be more effective for inducing such improvements rather than daily energy restriction due to an overall reduction in energy intake [55,59,60]. One study, conducted in young (pre-menopausal) adult women, found that weight loss was similar but fasting insulin and insulin resistance were both reduced to a greater extent in the intermittent fasting compared with the daily caloric restriction group [60]. Benefits of an intermittent modified fast diet strategy have been postulated to extend to reducing cancer risk and increasing the healthy lifespan of adults [61]. To date, studies examining the effectiveness of intermittent modified fasting have not been conducted in youth and findings from adult studies may not be directly applicable to a child or adolescent population.

## 8. Discussion

This review highlights the importance of weight loss in children and adolescents with obesity at increased risk of T2DM, supporting an adult review which suggests that a 10% weight loss conveys an 80% reduction in the incidence of T2DM [62]. Beyond weight loss, various dietary patterns that alter the quantity and quality of carbohydrates are discussed.

Reducing the quantity of carbohydrates in the diet may be an important strategy for reducing the risk of T2DM in youth via demonstrated effects on fasting insulin, insulin resistance, and glycaemic status, irrespective of weight change (Table 3). This may be achieved by prescribing a diet that is explicitly reduced in carbohydrates, such as a very low-carbohydrate diet or a VLED, or by altering the types of foods consumed or the pattern of consumption, such as a lower-GI diet or an intermittent fasting diet, which ultimately reduce the glycaemic load of the diet.

A recent review of adult studies similarly reports the important role of reducing dietary carbohydrates for the treatment of T2DM [63]. In fact, that review strongly suggests reappraisal of dietary recommendations, and that a low-fat high-carbohydrate diet be implemented for T2DM prevention and treatment, presenting 12 points of strong evidence from various research studies for why a reduced carbohydrate diet would be advantageous. However, current guidelines supporting the prescription of a low-fat, high-carbohydrate diet are based on findings from the largest T2DM prevention studies in adults which demonstrate such a diet to be highly effective in the prevention of T2DM when compared with no treatment [19,20,21,22,23]. Studies of the same magnitude are yet to be conducted using a reduced carbohydrate diet intervention making it difficult for guideline committees to introduce reduced carbohydrate diets into recommendations.

It appears intuitive to place an individual with insulin resistance or pre-diabetes on a lower carbohydrate diet. Presumably, a high-carbohydrate diet in these individuals would put additional pressure on an already stressed system that would result in higher glucose and insulin levels throughout the day [26]. Ultimately, this may predispose individuals to further blood lipid abnormalities and hypertension, which increases the risk for future cardiovascular disease as well as T2DM.

There is evidence from adult studies to suggest that the greater the carbohydrate restriction, the greater the improvement in T2DM risk factors [64]. As previously described, increased-protein diets with a carbohydrate content of 35%–50% did not improve insulin resistance and/or glycaemic status in children and adolescents compared with a standard-protein diet with carbohydrate content ranging from 50% to 60%. This suggests that carbohydrate content may need to be below 35% of energy intake to convey a benefit.

One hypothesised mechanism for the effect of a reduced carbohydrate diet in the prevention of T2DM is the effect on hepatic lipid content. Studies in adolescents have demonstrated that independent of total and visceral fat, hepatic fat is associated with glucose dysregulation, insulin resistance, and the metabolic syndrome [65,66]. Accumulation of fat in the liver leads to an increase in fat delivery to all body tissues, including the pancreas, which affects the islet cells of the pancreas and eventually down-regulates beta cell function [67]. Reducing the glycaemic load of the diet may facilitate reductions in hepatic fat by reducing postprandial glucose and insulin levels, leading to less hepatic glucose absorption and reduced hepatic lipid accumulation [24,25,68].

Another mechanism may be a reduction in inflammation achieved by reducing carbohydrate intake. Specifically, in the DiOGenes study, a main finding in adult participants was a significant decrease in inflammatory marker high-sensitivity C-reactive protein in lower-GI diet groups only, independent of protein content and weight change [69]. This finding was proposed to be related to reductions in postprandial glucose levels achieved through the lower-GI diet, with glucose known to stimulate the expression of inflammatory genes by epigenetic mechanisms [70,71,72].

The process of ketosis, such as in a very low-carbohydrate diet or a VLED, may also affect changes in insulin resistance and/or glycaemic status. The cardio-metabolic benefits of a ketogenic diet have been hypothesised to be due to weight loss. However, in both adult and paediatric studies, the effect on cardio-metabolic outcomes has been demonstrated to be more pronounced in a ketogenic diet compared with other diets, even with similar weight loss [27,28,29,73]. Mechanisms are not well understood but ketogenic diets have been demonstrated to specifically alter gene expression which may directly affect insulin signalling, insulin sensitivity, and glucose regulation, independent of weight change [74,75,76]. Urine ketone bodies have been identified in participants in studies not aiming for ketosis nor prescribing dietary carbohydrate levels consistent with ketone body production, including in an intermittent fasting intervention [58,60,77]. This suggests that ketosis benefits may be attained even with the prescription of more moderate carbohydrate intake.

There are negative aspects associated with a reduced carbohydrate diet that should be considered when deciding what type of diet should be prescribed to a patient (Table 3). Following a very low-carbohydrate diet has been demonstrated to lead to increased feelings of fatigue in adult men [78]. We speculate that this could result in a reduced desire to complete physical activity; especially in youth who are overweight or obese. This should especially be considered in an individual who is, or plans to be, very active as part of their weight loss regimen. It is of additional concern due to the well-established benefit of ongoing physical activity on maintenance of weight loss.

A very low-carbohydrate diet may be difficult for some individuals to follow long-term due to the required restriction of carbohydrate foods. While restricting or disallowing certain foods early on may assist in adherence due to limited food choices, this same aspect is likely to lead to boredom and may result in an inability to continue following the diet causing possible weight regain. This aspect is supported by the finding from our systematic review in youth that, short-term, a very low-carbohydrate diet led to greater weight loss compared with a high-carbohydrate diet, but longer-term weight loss was not significantly different, possibly due to eventual migration to a more typical carbohydrate intake [12].

Another issue is that restricting carbohydrates in the diet, without a sufficient increase in vegetable consumption, reduces the intake of nutrients obtained from high quality carbohydrates, particularly fibre and phytochemicals. A reduction in fibre intake in particular may increase the risk for gastrointestinal tract disorders. However, foods that contain such nutrients, such as whole grains, legumes, fruits, and vegetables, are typically lacking in a Western diet. Similarly, consuming lower-GI foods does not necessarily translate to consumption of healthier foods, with some ice cream, cakes, and potato crisps considered to be lower-GI. Furthermore, the GI of a food can vary between individuals and may not be directly applicable to children and adolescents at increased risk of T2DM [79].

An additional challenge is the difficulty defining which children and adolescents are at greatest risk of developing T2DM. For the purpose of this review we have widely defined youth with obesity as being at increased risk. However, those with insulin resistance and/or pre-diabetes may be assumed to be at greater risk. This is based on research that proposes that the transition from insulin resistance to pre-diabetes and type 2 diabetes occurs quite rapidly in youth [80]. However, more research is needed in this area to determine the speed and nature of this progression. Furthermore, the inconsistencies in reported findings could, in part, be due to the differences in the genetic background of individuals or populations in the included studies. However, this was not explored in the present review.

More high quality intervention studies are needed in youth to determine the association between carbohydrate intake and reduced risk of developing T2DM. However, if an association becomes clear, there appears to be several diet strategies that could be utilised to achieve a diet reduced in carbohydrates, including a very low-carbohydrate diet, a VLED, a lower-GI diet, and an intermittent fasting diet. This array of dietary options enables clinicians to offer a number of diet strategies to youth at increased risk of developing T2DM. Hence, diets may be personalised depending on patient preference and suitability.

## 9. Conclusions

Dietary research conducted in youth that measure T2DM risk factors and that compare macronutrient distributions and/or dietary patterns is limited. However, from the current body of evidence it appears that a diet that is lower in carbohydrates, irrespective of dietary strategy, may be particularly beneficial for improving risk factors for T2DM in youth.

## Figures and Tables

**Table 1:** Classification of diets based on carbohydrate content (modified from Liebman, 2014 [26]).

Carbohydrate Diet Classification	Amount of Carbohydrate	Example of Dietary Pattern
Typical/high-carbohydrate diets	45%–65% of total calories	Low-fat diet, STOP/Traffic light diet, Standard-protein diet, lower-GI diet
Moderately restricted carbohydrate diets	26%–44% of total calories	Intermittent fasting diet, increased-protein diet
Low-carbohydrate diets	51–130 g/day (or approximately 16%–26% of calories of a 2000 calorie diet)	Low-carbohydrate diet, Paleo style diet
Very low-carbohydrate diets [27,28,29]	Typically 20–50 g/day or 5%–15% of total calories	Very low-carbohydrate diet, very low-energy diet, Atkins diet

**Table 2:** Findings from studies examining the effect of various dietary patterns on type 2 diabetes risk factors in youth.

Dietary Patterns	Studies	Weight Outcomes	Other Outcomes
Very low-carbohydrate vs. low-fat diet	Gow et al., 2014 [12] ^1^	Possible short-term benefit of very low-carbohydrate diet	3 studies from review [27,28,29] report greater benefit of very low-carbohydrate diet for improving insulin resistance
Increased-protein vs. standard-protein diet	Gow et al., 2014 [12] ^1^; Garnett et al., 2014 [31] ^2^; Truby et al., 2016 [32] ^2^	No differences observed between groups	No differences observed between groups
Lower vs. higher glycemic index diet	Parillo et al., 2012 [34] ^2^; Iannuzzi et al., 2009 [35] ^2^; Joslowski et al., 2015 [36] ^2^; Damsgaard et al., 2013 [37] ^2^	2 studies [34,36] report significant benefit of lower glycemic index	3 studies [34,35,37] report greater benefit of lower glycemic index for improving insulin resistance
Very low-energy diet vs. low-fat diet	Figueroa-Colon et al., 1993 [38] ^2^; Berkowitz et al., 2011 [39] ^2^	Greater short-term weight loss and preservation of lean body mass in very low-energy diet [38,39]	No differences between intervention groups reported to date
Intermittent modified fasting	N/A	N/A	N/A

^1^ Systematic review including seven trials comparing a very low-carbohydrate to a low-fat diet and six studies comparing an increased-protein to a standard-protein diet; ^2^ Randomised controlled trial.

**Table 3:** Pros and cons of reducing carbohydrate in the diet.

Pros	Cons
Improved fasting insulin, insulin resistance and glycaemic status, irrespective of weight change	
Can be achieved via explicit carbohydrate reduction or altering dietary pattern	Increased fatigue could result in reduced desire to complete physical activity
Facilitates reduction in hepatic fat	May be difficult to follow long-term due to the required carbohydrate restriction
Facilitates reduction in inflammation	Reduced intake of fibre and phytochemicals if vegetable intake not suitably increased
Several diet strategies available to achieve reduced carbohydrate allowing individualisation of the diet to the patient	More research needed to support their use in youth
Greater short-term weight loss	
